# Wireless Passive Sensor Technology through Electrically Conductive Media over an Acoustic Channel

**DOI:** 10.3390/s23042043

**Published:** 2023-02-11

**Authors:** Thomas Schaechtle, Taimur Aftab, Leonhard M. Reindl, Stefan J. Rupitsch

**Affiliations:** Laboratory for Electrical Instrumentation and Embedded Systems, University of Freiburg, 79110 Freiburg, Germany

**Keywords:** passive sensor technology, resonant sensor, acoustic communication channel, wireless readout, harsh environment, chipless sensor

## Abstract

Hydrogen-based technologies provide a potential route to more climate-friendly mobility in the automotive and aviation industries. High-pressure tanks consisting of carbon-fiber-reinforced polymers (CFRPs) are exploited for the storage of compressed hydrogen and have to be monitored for safe and long-term operation. Since neither wired sensors nor wireless radio technology can be used inside these tanks, acoustic communication through the hull of the tank has been the subject of research in recent years. In this paper, we present for the first time a passive wireless sensor technology exploiting an ultrasonic communication channel through an electrically conductive transmission medium with an analog resonant sensor featuring a high quality factor. The instrumentation system comprised a readout unit outside and a passive sensor node inside the tank, coupled with geometrically opposing electromechanical transducers. The readout unit wirelessly excited a resonant sensor, whose temperature-dependent resonance frequency was extracted from the backscattered signal. This paper provides a description of the underlying passive sensor technology and characterizes the electric impedances and acoustic transmission as an electrical 2-Port to design a functional measurement setup. We demonstrated a wireless temperature measurement through a 10 mm CFRP plate in its full operable temperature range from −40 to 110 °C with a resolution of less than 1 mK.

## 1. Introduction

**Figure 1:**
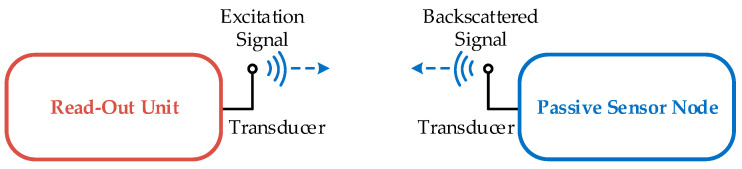
Schematic of a wireless passive sensor system: A readout unit sends out an excitation signal via a transducer into a wireless channel to a passive sensor node. There, this signal is picked up with the help of the transducer and stored in a high-Q resonator. When the excitation signal is turned off, the resonator rings out with its natural frequency and sends out a backscattered signal, which is picked up by the readout unit and evaluated.

Wireless passive sensor technology is an established instrumentation technique for environments where conventional battery-based radio technology cannot be operated or can only be implemented with extensive efforts. The technology is based on the wireless readout of a battery- and chip-free sensor node, which is referred to as a passive device. The instrumentation system consists of a readout unit and a passive sensor node, which are connected via a transducer to a wireless link, as seen in Figure 1.

The sensor node’s key component is a resonator with a high quality factor (*Q*). Its high-Q oscillation functions as a means of energy storage and additionally contains the information to be measured. In order to wirelessly poll the information, the resonator is excited by an excitation signal from the readout unit. The resonator vibrates in a forced oscillation and, when released, it vibrates exponentially, decaying at its natural frequency, provided that the natural frequency is within the spectrum of the excitation. A part of the decaying signal is backscattered and can be captured and analyzed by the readout unit. Consequently, if the resonance frequency is designed to be dependent on a physical quantity, this quantity can be wirelessly determined in the readout unit.

In the literature, LC resonators [1], ceramic dielectric resonators [2], and surface acoustic wave (SAW) resonators [3] have been investigated as resonant sensors in passive wireless sensor technologies to measure temperature, pressure, torque, and acceleration along with other physical parameters [4]. Due to the low complexity of the sensor node and the operability without a battery, this instrumentation technique is considered to be maintenance-free, robust, and therefore applicable in harsh environments. In all the publications mentioned above, an electromagnetic or inductive transmission was used to read out the passive resonant sensor. In applications with an electrically conductive medium within the transmission path or surrounding the sensor node, however, these transmissions are significantly attenuated and, therefore, cannot be used.

In contrast to electromagnetic or inductive transmission, acoustic waves propagate well through liquid or solid media, independent from their electrical conductivity. Acoustic wave propagation is exploited in numerous fields, such as diagnostic and therapeutic ultrasound for medical applications [5], non-destructive testing [6], indoor localization [7], and electrical energy transfer [8]. Ultrasonic waves in the low kHz range are utilized when mechanical or electrical energy is transferred with high power levels or over long distances, as in ultrasonic cleaning baths or underwater sonar. For medical imaging and non-destructive testing, frequencies of around 1 MHz up to 50 MHz are typically exploited to achieve high spatial resolution. The applied frequencies are chosen, to a great extent, based on the attenuation of ultrasonic waves due to inner friction, which increases drastically with higher frequencies [9] (p. 38) [10].

Various systems and methods for acoustic energy transfer (AET) or acoustic communication through liquids or solids in the frequency range of 20 kHz to a few MHz with power levels from µW to kW have been reported in the literature, with a typical configuration of two coaxially coupled transducers [11,12,13,14,15]. In the case of acoustic energy transfer, the electrical power is sent from a transmitter to a receiver using acoustic waves in the transmission medium, such as a metal or biological tissue. Efficiencies of 40% through animal tissues and 88% through a titanium plate have been reported [12,13,14].

Currently researched systems simultaneously transmit acoustic energy and data to allow the monitoring of process parameters through electrically conductive barriers. The sensor nodes typically comprise an electromechanical transducer, a rectifier with a battery management system and secondary battery, amplifiers and filter circuitries, modulation and demodulation modules, digital logic, and peripheral sensors [15,16].

Active systems exploiting a wireless acoustic channel have demonstrated the ability to transmit both energy and data over long ranges with high data transmission rates. The readout range is limited by the distance for which the rectifier circuit in the sensor node stops to supply the active circuit. The range of applications is limited to environments where semiconductor circuits can be operated.

Higher readout ranges and an extended field of operations might be achieved in systems exploiting a wireless acoustic channel by using a passive sensor node. The readout range for a wireless passive sensor node is limited by the distance, where the backscattered signal becomes undetectable due to noise. Since neither semiconductor devices nor batteries are required for operation, the range of applications is only limited to environments where the resonator and transducer can be operated. Thus, a passive sensor node could allow operation in extremely harsh environments.

Therefore, in this study, we proposed a passive wireless sensors system with an acoustic communication channel, combining the advantages of acoustic transmission through an electrically conductive transmission medium and the wireless passive sensor technique. This resulted in a wireless, maintenance-free, robust, and small-scale instrumentation technology with the potential to operate inside closed and electrically conductive containers under harsh environmental conditions. Such environmental conditions include aircraft wings and composite overwrapped pressure vessels (COPVs), in which high pressures and cryogenic temperatures dominate, as identified in [17,18]. Based on a recently published comprehensive review on passive resonant sensors [19], and to the extent of our knowledge, no wireless passive sensor through an electrically conductive transmission medium over an acoustic channel has been reported in the literature. Preliminary experiments were carried out in [20] with airborne communication and a basic measurement setup. This contribution represents an extension to [21], with further characterization of the components, a detailed description of the signal processing concept, and an extensive evaluation of the performance of the instrumentation technique.

## 2. Concept behind the Acoustic Passive Wireless Sensor Technology

**Figure 2:**
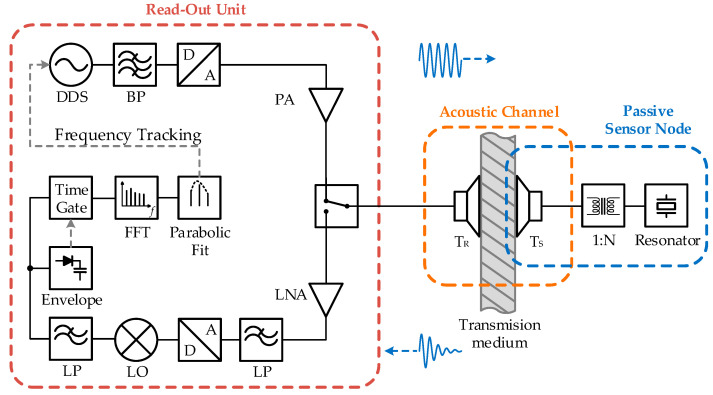
Block diagram of the wireless passive sensor technique using an acoustic channel for the connection between the readout unit and the passive sensor node.

The instrumentation for a wireless passive sensor system comprises a readout unit and a passive sensor node, as illustrated in Figure 1 and the implemented block diagram in Figure 2. The functional modules for this acoustic sensor system are equivalent to radio-based systems, as shown, for example, in [3,4,22]. However, in the case of an acoustic channel, the antennas are exchanged for the electromechanical transducers $T_{R}$ and $T_{S}$ of the readout unit and the sensor node, respectively.

For the selection of the operation frequency, the acoustic attenuation in the transmission medium is one of the most relevant parameters. The acoustic attenuation $\alpha_{d}$ is attributed to dislocation damping and can be described in several solid media for a frequency range of up to 15 MHz, typically with a linear frequency dependency, by:

(1) $$\alpha_{d}=C_{d}\cdot f\;,$$

with the material constant for attenuation $C_{d}$ in dB/m/MHz and the frequency f in MHz. A comprehensive study of acoustic damping mechanisms in various materials, such as metals, ceramics, CFRPs, and other organic materials, with an extensive list of material parameters is provided in [10]. The material constants were characterized by large inaccuracies; for example, for temperature-treated aluminum AL 6061, the values ranged from 2.9 to 14.4 dB/m/MHz, and for different types of CFRP, they ranged from 148 to 1733 dB/m/MHz in the direction of the surface normal [10].

Therefore, an operation frequency for the instrumentation system in a low MHz range was suggested. Hence, time-domain sampling could be performed by the instrumentation system with moderate hardware requirements. The passive sensor node was read out with a pulse-echo method, whereby an excitation pulse was transmitted from the readout unit, and the sensor information was extracted from a backscattered echo from the passive sensor node. If the signal-to-noise ratio (SNR) of the backscattered signal was high, this method allowed high update rates for the measurement, which are especially important for low-frequency resonators with high quality factors above 10,000 [4].

A sinusoidal excitation pulse of the readout frequency $f_{R}$ and its duration $t_{P}$ was generated by direct digital synthesis (DDS) and digital-to-analog conversion. The sinusoidal signal with its nearly rectangular pulse shape in the time domain resulted in a narrowband sinc signal, centered at $f_{R}$ in the frequency domain. The excitation pulse was amplified to drive the electromechanical transducer $T_{R}$ at a higher power level, increasing the signal strength received by the sensor node and subsequently the backscattered echo.

The excitation pulse was converted into an acoustic wave by the electromechanical transducer $T_{R}$ and transmitted into the transmission medium. A fraction of the incident acoustic wave at the sensor side was converted back into an electrical signal by the transducer $T_{S}$ and excited the high-Q resonator in the passive sensor node. If the resonance frequency $f_{0}$ was within the spectrum of the excitation pulse, the resonator was driven into forced oscillation at frequency $f_{R}$, and energy was stored in weakly damped oscillation.

When the excitation pulse ended, the forced oscillation of the resonator at frequency $f_{R}$ was converted to free oscillation at the resonance frequency $f_{0}$ with an exponentially decaying amplitude A(t) of:

(2) $$A\left(t\right)=A_{0}\cdot e^{-t/\tau }\;,$$

with the maximum amplitude $A_{0}$ after turning off the excitation pulse at time t=0. The time for excitation and decaying was given by the decay constant τ, which depended on the quality factor *Q* of the resonator:

(3) $$\tau =\frac{Q}{\pi \;\cdot f_{0}\;}\;.$$

Within an interval of 3 to 5 τ, 95 to 99% of the maximum obtainable amplitude $A_{0}$ was stored in the resonator during the pulse duration and dissipated in the ring out during the echo duration [4].

The quality factor *Q* could be equivalently derived in the frequency domain as follows:

(4) $$Q=\frac{f_{0}}{B}\;,$$

where *B* denotes the −3 dB bandwidth at resonance frequency $f_{0}$ [23,24].

Part of the exponentially decaying signal stored in the resonator was converted back into an acoustic signal via the transducer $T_{S}$ and radiated into the acoustic transmission medium, resulting in a backscattered signal. A small fraction of this signal was then picked up by the transducer $T_{R}$ in the readout unit. After electromechanical conversion, the signal was low-pass filtered and digitized. In the digital domain, the data were down-converted to baseband with the local oscillator (LO), decimated, and low-pass filtered. In this way, we could reduce the amount of data and the computational effort to allow for a fast measurement update rate.

The envelope of this signal was then used for edge detection between two sequential pulses. This provided a stable and accurate time synchronization between the data streams of the transmitted excitation pulse and the received echo signal. This was required for the separation of the decaying echo signal of the resonator from undesired reflections generated by the excitation pulse in the acoustic channel. Due to the high quality factor *Q* of the resonator, the echo signal could be detected over a longer time interval than these reflections, and separation could be carried out in the time domain with a time gate. To estimate the resonance frequency $f_{0}$ of the resonator and, therefore, the sensor information, we applied a Tukey window to the time-gated signal and subsequently implemented fast Fourier transformation (FFT).

In the frequency domain, the maximum amplitude with a sufficient difference from the noise floor could be identified as the resonance frequency $f_{0}$. The Fourier transform of an exponential decaying signal results in a Lorenz function, which can be approximated around the maximum with a parabola. To enhance the frequency resolution of the discrete frequencies of the FFT, the frequency bins around the resonance were approximated with a parabolic fit. The maximum value of this parabola yielded the resonance frequency $f_{0}$ of the resonator with a higher resolution. An enhancement in the resolution by a factor of 10 has been reported with this technique [4,25]. To achieve a maximum signal strength for the backscattered echo at a possibly varying resonance frequency, the subsequent pulse was excited at the determined resonance frequency. This frequency tracking method maintained the narrowband excitation spectrum around the resonance frequency.

## 3. System Design

To investigate the performance of the passive sensor system operating wirelessly with acoustic transmission, a temperature measurement setup inside a hydrogen pressure tank consisting of carbon-fiber-reinforced polymers (CFRPs) was selected. The key element of the instrumentation system was the resonant sensor, which stored the sensor information in its resonance frequency. In principle, different types of resonators could be electrically coupled to the acoustic channel. However, due to the linear increase in the acoustic attenuation in the transmission medium, the selection of the operation frequency was critical for the first demonstration of the passive sensor system. SAW resonators have been widely used in passive sensor systems, as they feature high quality factors [3,4]. As a matter of fact, they are only available with typical resonance frequencies from 50 MHz up to 3 GHz [26] (p. 1386). With the lowest acoustic attenuation in the CFRP being 148 dB/m/MHz, and the lowest resonance frequency being 50 MHz, this resulted in an acoustic attenuation of at least 74 dB per path at a distance of 10 mm, assuming a further linear frequency dependency as described in (1).

Passive LC resonators combining inductive and capacitive elements can be developed as resonant sensors in the range of several kHz to several hundred MHz with quality factors up to 100, as comprehensively reviewed in [27]. According to (3), this would result in a decay time of less than 300 µs for frequencies above 100 kHz, which may not be sufficient for time-domain separation with environmental echoes in an acoustic channel.

**Figure 3:**
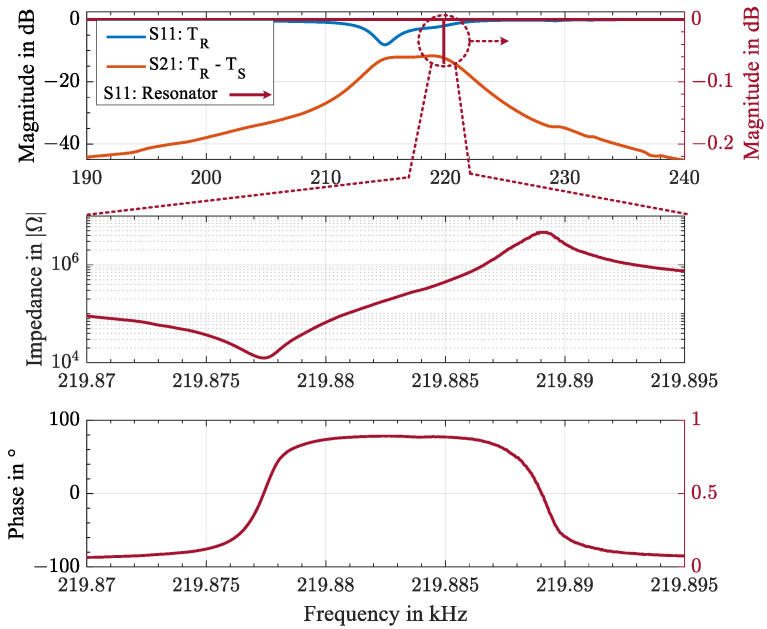
Electrical characterization of the piezoelectric transducer $T_{R}$ and $T_{S}$ together with the high-Q resonator: input return loss $S_{11}$ of the piezoelectric disc transducer $T_{R}$ in blue, insertion loss $S_{21}$ of $T_{R}$ and $T_{S}$ through the 10 mm CFRP plate in orange, and $S_{11}$ of the high-Q resonator in red on the right axis (**top**). The two lower graphs show a 25 Hz zoomed-in frequency range of the impedance according to the amplitude and phase of the high-Q resonator at room temperature (**middle** and **bottom**).

A tuning fork resonator provides suitable resonance frequencies with high quality factors. Therefore, a TS4 piezoelectric tuning fork resonator produced by Statek Corp (Orange, CA, USA) was selected as the high-Q element and sensor. The tuning fork resonator was manufactured from quartz, with a temperature-sensitive crystal cutting angle. The tuning fork resonator featured a fundamental torsional mode at 219.875 kHz at room temperature. On the one hand, this low frequency ensured minimal acoustic attenuation in the transmission medium; however, on the other hand, it enlarged the geometric dimensions of the electromechanical transducer considerably. Furthermore, the drive level of a tuning fork resonator is low, and therefore the energy stored in the vibration is limited. To characterize the high-Q tuning fork resonator, the electric impedance was measured with a high-precision impedance analyzer Hewlett Packard HP4294A. It was converted to a return loss $S_{11}$, as shown in the first graph in Figure 3. The second and third graphs depict the impedance in terms of amplitude and phase in a zoomed-in frequency range of 25 Hz at room temperature.

To excite the resonator over the acoustic channel, the transfer characteristics of the electromechanical transducers had to fit the resonator’s frequency. We utilized a pair of piezoelectric discs from Steiner & Martins Inc. (Davenport, FL, USA) with a diameter of 10 mm, a thickness of 2 mm, and a wraparound electrode. This piezoelectric disc exhibited a fundamental radial mode at 215 kHz ± 5 kHz. The radial mode allowed for significantly smaller dimensions while maintaining good coupling and the focusing of the main lobe of the longitudinal wave propagation at a given operation frequency [28,29].

The scatter parameters of the piezoelectric discs mounted with a silicon rubber adhesive on two opposite sides of the CFRP plate were measured using the vector network analyzer R&S ZVL6 to characterize the electrical characteristics of the acoustic channel. Figure 3 displays in the upper graph the input return losses $S_{11}$ and $S_{22}$ of the piezoelectric disc transducers $T_{R}$ and $T_{S}$, respectively, in blue and the insertion loss $S_{21}$ in orange through the 10 mm CFRP plate. The minimum insertion loss of the radial mode with $S_{21}=-11.7$ dB appeared at 218.85 kHz. The −3 dB transmission band ranged from 215 kHz to 221 kHz. Since the setup was a reciprocal network, $S_{11}=S_{22}$ and $S_{21}=S_{12}$ held.

To match the electric impedance of the piezoelectric disc transducer to the impedance of the tuning fork resonator, a transformer was used whose turns ratio allowed the transformation of the impedance [30,31]. The impedance of the piezoelectric disc transducer was calculated from the measured input return loss with $Z_{0}=50\;\Omega$.

**Figure 4:**
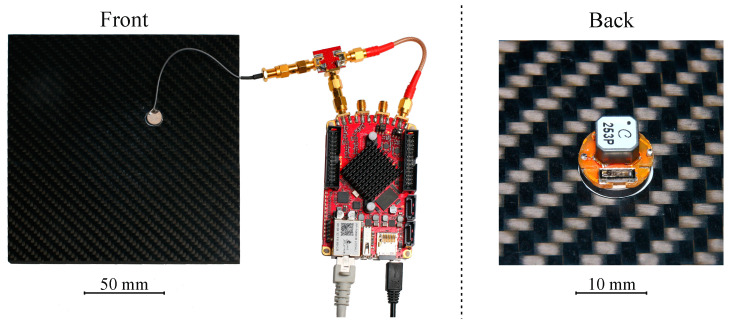
Demonstration measurement setup with outer piezoelectric disc transducer $T_{R}$ and the readout unit (**left**) and the inner passive sensor node with piezoelectric transducer $T_{S}$ coaxially coupled to $T_{R}$ through a 10 mm thick CFRP plate with the high-Q resonator and impedance matching mounted on a printed circuit board (**right**).

We implemented a measurement system as shown in Figure 4. The software-defined radio STEMlab 125-14 from Red Pitaya generated an excitation signal set to 1 $V_{pp}$, performed time-domain sampling at 1 MHz, down-converted the signal to baseband, and streamed it to a computer for further signal processing. To enable the tracking of the excitation to the resonance frequency for fast temperature changes, the measurement repetition rate was set to 4 Hz, which resulted in a total duration of 250 ms for the pulse and echo interval.

The geometric dimensions of the passive sensor node corresponded to a cylinder with a diameter of 10 mm and a height of 6 mm. The dimensions of existing acoustically powered and readout active sensor systems are mostly not provided. In [16], the sensor node consisted of a printed circuit board with a densely assembled and complex integrated circuitry, presenting an area of 31.7 by 31.7 mm^2^ and an estimated height of 5 mm. The dimensions and complexity of this system increased the challenges for integration into a COPV at very high pressures.

**Figure 5:**
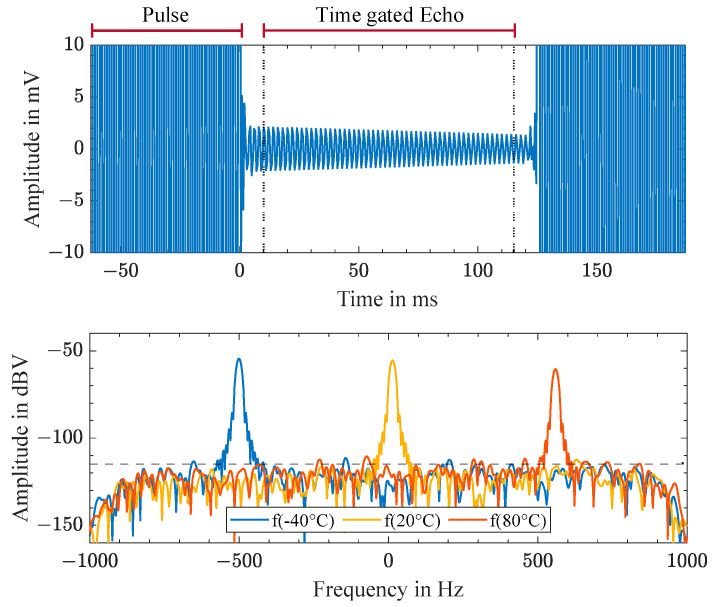
Sampled time-domain signal after down-conversion for a high-Q resonator at −40 °C with excitation pulse at t<0 ms and backscattered echo starting at t=0 ms (**top**). Fourier transform of the time-gated echo signal between 10 ms and 115 ms for three different temperatures of the tuning fork resonator: in blue for −40 °C, in yellow for +20 °C, and in orange for +80 °C (**bottom**).

Figure 5 depicts in the upper graph the sampled signals after down conversion in the time domain for a resonator at −40 °C. The excitation pulse could be seen up to t<0 ms and was turned off at t=0 ms. After some post-pulse oscillations, due to relaxing effects in the power amplifier and in the transducer disc, and due to reflections of the acoustic waves in the CFRP plate, the backscattered echo of the tuning fork remained. After 125 ms, the next excitation pulse occurred. The backscattered echo was cut out between 10 ms and 115 ms using a Tukey window function and was subsequently Fourier-transformed. The lower graph of Figure 5 shows in the frequency domain the Fourier transform of the time-gated echo signals for three different temperatures at the tuning fork resonator, alongside the temperature-dependent resonance frequency.

**Figure 6:**
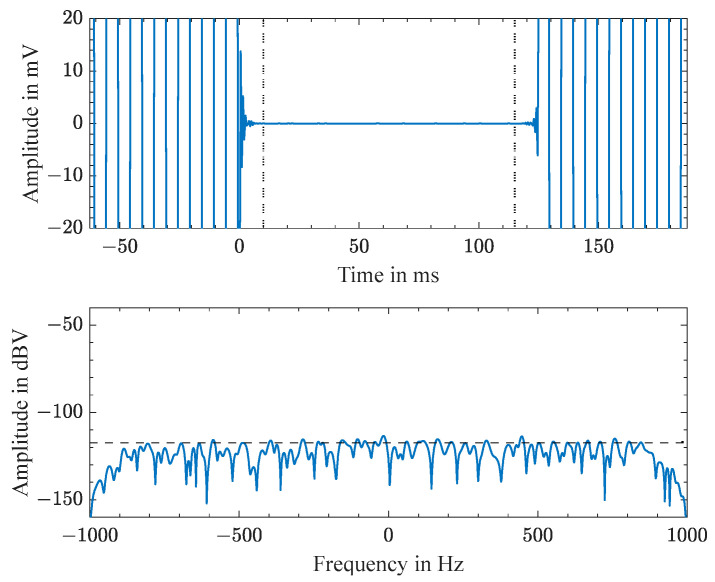
Measured time-domain signal without attached resonator (**top**) and corresponding time-gated echo signal in the frequency domain (**bottom**).

When measuring a pulse and echo without a high-Q resonator attached, no backscattered signal was detectable in the time-gated echo signal, as can be seen in Figure 6. The upper graph shows the sampled signals after down-conversion in the time domain, and the lower graph shows in the frequency domain the Fourier transform of the time-gated echo signal. The noise floor with the side lobes of the window function were below −110 dBV.

## 4. Results

**Figure 7:**
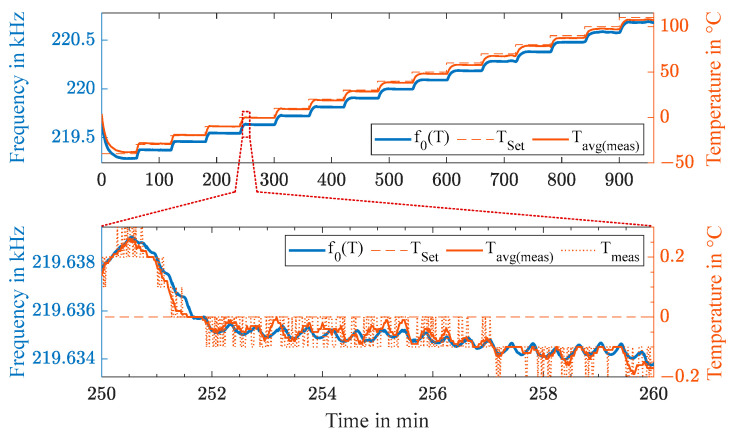
Measurement of the resonance frequency over a temperature range from −40 to 110 °C through a 10 mm thick CFRP plate (**top**) with an enlarged section at 0 °C (**bottom**).

With the experimental instrumentation and signal processing concept presented in Section 2 and Section 3, we wirelessly measured the characteristic resonance frequency of the passive tuning fork resonator over an acoustic channel through a 10 mm thick CFRP plate for a temperature profile ranging from −40 to +110 °C with a stepwise increase of 10 °C over 16 h. The sensor node with its resonator, piezoelectric transducer $T_{S}$, and electrical matching network was placed inside a climate chamber. The 10 mm thick CFRP plate with a rubber sealing covered the opening of the climate chamber, upon which the transducer $T_{R}$ and the readout unit were placed outside. Thus, realistic operating conditions were established with temperature differences between $T_{R}$ and $T_{S}$. Figure 7 shows in the upper graph the resonance frequency of the acoustic passive sensor node, which was wirelessly measured using acoustic waves. The measured frequency is shown in blue, the set temperature is indicated by a dashed line, and the average of the measured temperature is illustrated in orange. The lower graph depicts a zoomed-in portion at the temperature of 0 °C, where the temperature control of the climate chamber could be obtained as five times the averaged reference temperature $T_{\mathrm{avg}\left(\mathrm{meas}\right)}$.

**Figure 8:**
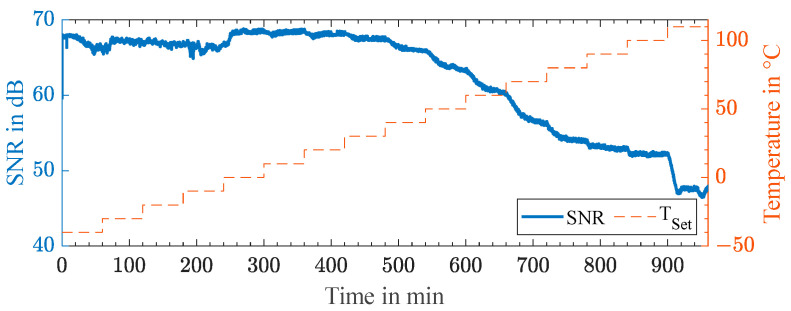
Signal-to-noise ratio in the measured resonance frequency shown in Figure 7.

The resonator had a quadratic response to temperature T. The resonance frequency f(T) could be fitted by:

(5) $$f\left(T\right)=\left(219632.4+8.87\cdot T+0.0057\cdot T^{2}\right)\;\mathrm{Hz},$$

with a root mean square error of 1.664 Hz for 220 000 points. For a total temperature change of 150 K, a shift in frequency of 1.397 kHz was observed, yielding a linearized sensitivity of 9.32 Hz/K with a root mean square error of 10.14 Hz. The precision of the wireless measurement was mainly determined by the signal-to-noise ratio when evaluating the resonance frequency of the tuning fork resonator. Figure 8 displays the signal-to-noise ratio in the measurement of the resonance frequencies shown in Figure 7. For the wireless passive sensor technique, an uncommonly high signal-to-noise ratio of 68 to 48 dB was observed over the entire temperature range. The pronounced decrease in the signal-to-noise ratio at temperatures above 40 °C could be attributed to the shift in the resonance frequency, distancing it from the minimum insertion loss $S_{21}$ of the transducers $T_{R}$ and $T_{S}$ in Figure 3 with a decrease of 1.1 dB from 220 to 220.5 kHz. Additionally, the decrease might have been due to the softening of the adhesive layer or the epoxy of the CFRP plate itself as it approached its maximum operating temperature. This resulted in greater attenuation and reflections in the acoustic channel. Furthermore, the quality factor of the tuning fork resonator decreased at higher temperatures. The degradation was found to be reversible in the subsequent cooling phase. In general, a high SNR was observed over the entire temperature range, allowing continuous frequency tracking. For an optimized SNR, the pulse and echo interval duration and the backscattered energy from the resonator must be investigated further. For this purpose, the viscoelastic losses in the resonator, studied in [32], need to be adapted for temperature-sensitive quartz crystal cutting.

The enlarged section in the lower graph of Figure 7 shows for a time interval of 10 minutes the set temperature $T_{\mathrm{Set}}$ of the climate chamber, the measured reference temperature $T_{\mathrm{meas}}$ of the thermocouple with a resolution of 0.1 °C, and an averaged value $T_{\mathrm{avg}\left(\mathrm{meas}\right)}$ over five samples. The accuracy of the temperature control of the climate chamber within a time interval of 30 s is indicated clearly by the data. Thus, the temperature control of the climate chamber provided no sufficiently stable temperature basis for a detailed statistical evaluation of the wireless measurement’s precision.

**Figure 9:**
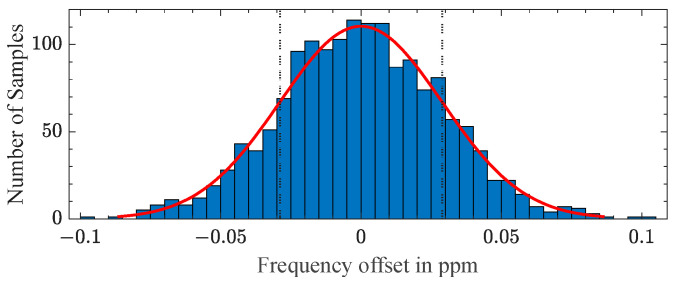
Histogram of 1600 temperature measurements at a constant temperature with a normal distribution fit.

Therefore, the temperature sensor was wirelessly measured in a thermally isolated, inactive climate chamber to investigate the precision of the wireless measurement system at a non-regulated, presumably constant temperature. The mean value µ of the resonance frequency was found to be 219,849.43 Hz with a standard deviation of 6 mHz, corresponding to 0.029 ppm, as shown in Figure 9. The 1600 measurement samples are shown in a histogram, together with a fitted normal distribution. The goodness-of-fit to the normal distribution was confirmed with a 0.01% significance level by a chi-square test, which further indicated a constant temperature during the sample duration.

The precision of the measurement could be enhanced by the averaging of the samples. Averaging over four samples led to a standard deviation of 3.2 mHz at a measurement duration of 1 s. The obtained sensitivity shown in Figure 7 and the standard deviation shown in Figure 9 demonstrated the short time precision of the wireless temperature measurement with a resolution of less than 1 mK. To transform this short time precision into robust accuracy, a careful calibration of the system would be needed, together with further investigations into the clock stability of the readout unit over time and temperature changes and the aging of the resonant sensor.

**Figure 10:**
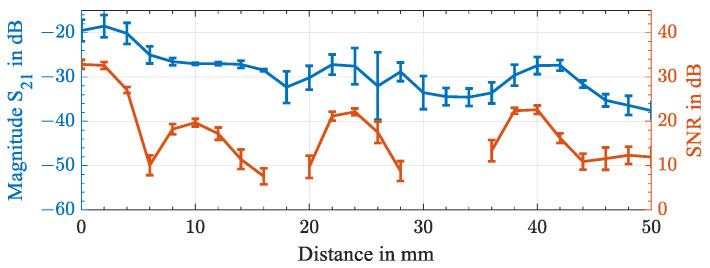
Minimum insertion loss $S_{21}$ of the piezoelectric transducers $T_{R}$ and $T_{S}$ in blue and the signal-to-noise ratio of a wirelessly read out resonance frequency at room temperature in orange through a 15 mm thick CFRP plate with the axial displacement of the transducers ranging from 0 to 50 mm.

Additionally, the effects of an axial offset of the two transducers are shown in Figure 10. Here, one transducer was bonded to a 15 mm thick CFRP plate with silicone rubber, and the second transducer was acoustically coupled with non-permanent glycerin and placed 2 mm away from its previous position for each measurement. We compared a measurement of the insertion loss $S_{21}$ between the two transducers to the signal-to-noise ratio of the wireless temperature measurement through the 15 mm thick CFRP plate. For the measurement of the scattering parameter, the position of the transducer was altered four times between 0 and 50 mm, and the minimum insertion loss with the standard deviation was obtained. The mean frequency with the minimum insertion loss over all measurements was found to be 216.4 kHz ± 340 Hz. It should be noted that the 5 mm thicker plate corresponded to an attenuation of 6 dB at a 0 mm axial offset.

To obtain the signal-to-noise ratio, the passive sensor node was positioned 2 mm further away for each iteration, and the resonance frequency was wirelessly read out through the 15 mm thick CFRP plate at room temperature. The signal-to-noise ratio was averaged over 40 measurements. The measurement of the temperature was successful up to an axial distance of 50 mm. The tested distance was limited by the dimensions of the CFRP plate. Constructive and destructive interference was present. At positions where the SNR was below 5 dB, the resonance could not be tracked continuously, and no data point is given. With an increase in the transmitted power of the readout unit, the SNR could be improved, particularly at greater distances. However, the transmitted signal was limited by the present circuitry to an amplitude of 1 $V_{pp}$. For higher excitation amplitudes, the maximum drive power of the resonator needs to be considered. The measurement showed that a placement within a radius of 4 mm was not critical, which is favorable for the subsequent integration into a COPV.

## 5. Conclusions

In this study, we proposed a novel instrumentation technique to wirelessly read out a passive sensor node through an electrically conductive transmission medium. The presented instrumentation read out a wireless passive resonant sensor with a pulse-echo method over an acoustic channel, which made it the most promising wireless instrumentation system for operation within an electromagnetically shielded, harsh environment. Within the manuscript, a design approach combining wireless passive sensor technology with acoustic transmission was presented. The temperature-sensitive high-Q resonator and the acoustic transmission channel with electromechanical transducers were electrically characterized, a functional demonstrator was designed, and the performance was evaluated. Thus, a wireless temperature measurement through a 10 mm thick CFRP plate with a signal-to-noise ratio above 48 dB was demonstrated over the entire operational temperature range of the lightweight construction material, i.e., from −40 to 110 °C.

In future work, an analytical model will be derived with the aim of increasing the SNR. This could be achieved by optimizing the pulse and echo intervals and the electrical loading of the resonator. Secondly, application-related work will focus on the protection and installation of the sensor node inside a COPV with minimal impact on the acoustic transmission.

## Data Availability

The raw data of graphs and tables presented in this study are available on request from the corresponding author.
